# Supplementary material for: Spontaneous Emergence of Multicellular Heritability
Source: Genes (Basel). 2023 Aug 17;14(8):1635. doi: 10.3390/genes14081635 (PMC10454505; doi:10.3390/genes14081635)
Supplement: Supplementary file 1 [file genes-14-01635-s001.zip › genes-2487310-supplementary.pdf]

Supplementary material

Calculating heritability by partitioning variance.

We calculated broad-sense heritability as the correlation coefficient between parental cell phenotype α (cellular aspect ratio) and offspring cell phenotype α' for cellular aspect ratio, partitioning the phenotype into its key components (Fisher 1919):

$$H^2 \equiv Corr(\alpha, \alpha') = \frac{E[(\alpha - \overline{\alpha})(\alpha' - \overline{\alpha'})]}{Var(\alpha)} \tag{1}$$

Where:

$$\alpha = G + E + S \quad \alpha' = G + E' + S' \tag{2}$$

In this equation G is the genetic mean of aspect ratio, E are environmental effects with mean $\bar{E}$ and variance σ_E^2, and S is intrinsic variation in the expression of the trait, which we refer to as developmental noise. This term captures emergent variation in how cells develop (when referring to S, cell-level developmental noise), or in how groups of cells develop (when referring to S'). Cell-level heterogeneity is fairly straightforward: genetically identical cells reared in a common environment are never phenotypically identical. To explain group-level developmental noise more clearly, let us consider an example of a group-level trait that is affected by cellular interactions: cluster size. Cluster size is a function of cell shape, cell number, and cell arrangement in the cluster. Cell shape and cell number are cell-level traits that can be measured individually, but cell arrangement is a group-level trait that depends on how cells are positioned and oriented relative to each other. Cell arrangement can vary among clusters within a genotype due to cellular interactions with each other, and may have a stochastic component. In the snowflake yeast model system, this may arise as a consequence of the location and angle of cellular budding, which affects mechanical interactions among cells, the resulting strain accumulation from growth, and thus size. In yeast, bud site location is fundamentally stochastic, so multiple clonal clusters developing from single cells (all the same genotype) will have some variation in multicellular phenotype. Here we assume that S is a white noise, with mean zero and variance (σ_S^2). Prime terms refer to offspring.

Assuming no mutation, selection, or temporal correlation except for the same genotype between offspring and parents, then $\overline{\alpha} = \overline{\alpha'}$ and we can rewrite the numerator as:

$$E[(\alpha - \overline{\alpha})(\alpha' - \overline{\alpha})] = E[\alpha\alpha'] - \overline{\alpha}^2 = \sigma_G^2 \tag{3}$$

So the heritability of aspect ratio is equal to:

$$H_\alpha^2 = \frac{\sigma_G^2}{\sigma_G^2 + \sigma_S^2 + \sigma_E^2} \tag{4}$$

If N (number of cells at division) is a linear function of the mean of cellular aspect ratio, then N is also a linear function of G, because all the cells in a clonal cluster have the same genetic mean value for aspect ratio. But because developmental noise S is random and different for each cell, its expected value for all the cells in a cluster is zero. We also consider developmental noise at the cluster level, ϵ, which accounts for internally-generated (i.e., not environmental) variation in cluster phenotype. Finally, we use the proportionality constant a to relate the number of cells at division (N) to the sum of genetic mean (G), environmental effects (E), and developmental noise at the cluster level (ϵ). This is a scaling factor that converts the combined effect of these components into the number of cells at division.

So for N we have: $N = a(G + E + \epsilon)$, $\overline{N} = a\overline{G}$, $Var(N) = a^2(\sigma_G^2 + \frac{\sigma_E^2}{N} + \frac{\sigma_S^2}{N} + \sigma_\epsilon^2)$. Given these equations, the heritability of the number of cells at division in the simplest form is equal to:

$$H^2_N = \frac{\sigma^2_G}{\frac{\sigma^2_S}{N} + \frac{\sigma^2_E}{N} + \sigma^2_\epsilon + \sigma^2_G} \quad (5)$$

so the ratio of the heritability of the number of cells per cluster and cell size is equal to:

$$\frac{H^2_N}{H^2_\alpha} = \frac{\sigma^2_S + \sigma^2_E + \sigma^2_G}{\frac{\sigma^2_S}{N} + \frac{\sigma^2_E}{N} + \sigma^2_\epsilon + \sigma^2_G} \quad (6)$$

The heritability of the group-level trait will be higher than the cell-level trait when the ratio $\frac{H^2_N}{H^2_\alpha}$ is greater than 1. This happens when:

$$\sigma^2_S + \sigma^2_E + \sigma^2_G > \sigma^2_S/N + \sigma^2_E/N + \sigma^2_\epsilon + \sigma^2_G \quad (7)$$

Reordering the terms, we get:

$$(N-1)(\sigma^2_S + \sigma^2_E) > N\sigma^2_\epsilon \quad (8)$$

The heritability of the group-level trait will be higher when:

1) The group size (N) is larger: As the group size increases, the difference between N and (N-1) becomes less significant, and the effect of cell-level developmental noise (σ^2_S) and environmental variation (σ^2_E) is averaged out over a larger number of cells, reducing their impact on the group-level trait.

2) The sum of cell-level developmental noise and environmental variation ($\sigma^2_S + \sigma^2_E$) is large relative to the group-level developmental noise (σ^2_ϵ): If cell-level developmental noise and environmental variation are large compared to group-level developmental noise, the heritability of the group-level trait will be higher as the individual contributions of these factors are reduced through averaging over the group size.

Supplementary Figures

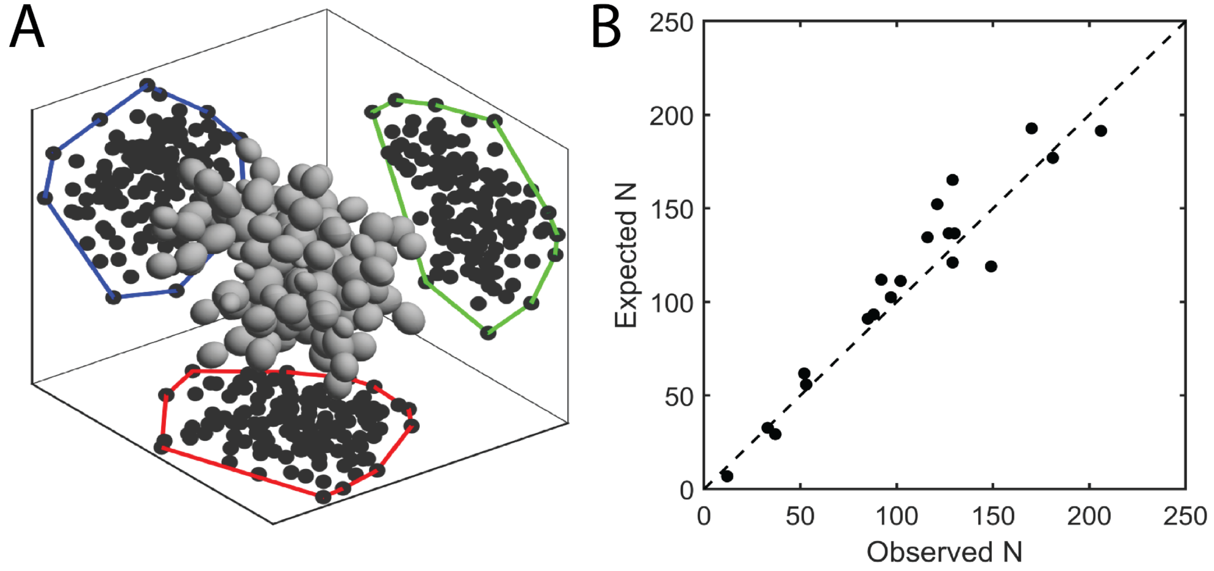

Figure S1. Estimating cell number from cross-sectional area, cell size, and packing fraction using Serial Block Face Scanning Electron Microscopy (SBF-SEM). To validate the method used to estimate cell number via time-lapse microscopy, we used previously published data of 20 clusters of snowflake yeast (ace2) sectioned using SBF-SEM with 50 nm z sections (Bozdag et al. 2021). This form of microscopy provides complete information about cell locations (x,y, and z) within the group. We then used these spatial coordinates to estimate a perceived cross-sectional area of the cluster that would be similar to a measurement obtained

from optical microscopy. We first collapsed all data onto the same (xy)-plane (i.e. removing z spatial information), and measured the area of the convex hull of those points. We also did this for the (xz)-plane, and the (yz)-plane (A), and averaged those three measurements to obtain a mean cross-sectional area. This measurement should be comparable to the 2D cross sectional area measured via light microscopy in the time lapse experiments. Separately, we calculated the average cell volume, and each cluster's packing fraction (B), from which we calculated an average packing fraction. We then estimated the volume of each cluster (V_{est}) using the formula $V_{est} = 4\pi/3\sqrt{Area/\pi}^3$, and computed the expected number of cells (N_{exp}) with the formula $N_{exp} = PackFrac * V_{est}/V_{cell}$, and compared it to the actual number of cells in the cluster. The figure displays a plot of the expected number of cells (N_{exp}) versus the observed number of cells (N_{obs}), with $y = x$ plotted as a dashed line. Our method accurately estimates the number of cells within the cluster, given knowledge about the 2D cross sectional area, cell size, and cell packing fraction ($R^2 = 0.903$, linear regression constrained to $y = x$.f)

Supplementary Movies

Movie S1. Growth and internal stress-mediated division of a snowflake yeast cluster (genotype *ace2*Δ *clb2*Δ). Sampling interval was 2.5 minutes, for a total observation time of 15 minutes.

Supplementary Tables

Table S1: Oligonucleotides used for strain construction

Oligo	Sequence
*clb2*Δ F	CCAAGAAGCCTTTTATTGATTACCCCCTCTCTCTCTTCATTGATCTTATAGatcgatgaattcgagctcg
*clb2*Δ R	GGACATTTATCGATTATCGTTTTAGATATTTTAAGCATCTGCCCCTCTTCgacatggaggcccagaatac
*akr1*Δ F	TCCGTTTCGTCTAGATAAAAAAACACTTCTTTGTTCAGAGTAGCTAATTGatcgatgaattcgagctcg
*akr1*Δ R	TGATAAAAGGCTAAAATATACAGTTTCTCCTAATGAAAACAACAAAATTTgacatggaggcccagaatac
*arp8*Δ F	TAAATTACTAGTCAATAGTACATAAATACAGGGATACAATCGCACCTAACatcgatgaattcgagctcg
*arp8*Δ R	TGCAAAGACCTTTCAGAAAAAAAGATAACAAAAACTTCCATATGCATATCgacatggaggcccagaatac

Table S2: Yeast strains

Oligo	Sequence
GOB8	*ace2*Δ:KanMX/*ace2*Δ:KanMX
AJB85	*ace2*Δ:KanMX/*ace2*Δ:KanMX, *clb2*Δ:hphNT1/*clb2*Δ:hphNT1
AJB132	*ace2*Δ:KanMX/*ace2*Δ:KanMX, *akr1*Δ:hphNT1/*akr1*Δ:hphNT1
AJB134	*ace2*Δ:KanMX/*ace2*Δ:KanMX, *arp8*Δ:hphNT1/*arp8*Δ:hphNT1

References

Bozdag, G. O., Libby, E., Pineau, R., Reinhard, C. T., and Ratcliff, W. C. (2021). Oxygen suppression of macroscopic multicellularity. *Nature Communications*, 12(1):2838.

Fisher, R. A. (1919). Xv.—the correlation between relatives on the supposition of mendelian inheritance. *Earth and Environmental Science Transactions of the Royal Society of Edinburgh*, 52(2):399–433.
